# A Systematic Review of the Time Series Studies Addressing the Endemic Risk of Acute Gastroenteritis According to Drinking Water Operation Conditions in Urban Areas of Developed Countries

**DOI:** 10.3390/ijerph15050867

**Published:** 2018-04-26

**Authors:** Pascal Beaudeau

**Affiliations:** Santé Publique France, 14 rue du Val-d’Osne, 94415 Saint-Maurice CEDEX, France; pascal.beaudeau@santepubliquefrance.fr; Tel.: +33-179-416-822

**Keywords:** acute gastroenteritis, risk, tap water, time series study, turbidity, urban area, water operation data

## Abstract

Time series studies (TSS) can be viewed as an inexpensive way to tackle the non-epidemic health risk from fecal pathogens in tap water in urban areas. Following the PRISMA recommendations, I reviewed TSS addressing the endemic risk of acute gastroenteritis risk according to drinking water operation conditions in urban areas of developed countries. Eighteen studies were included, covering 17 urban sites (seven in North-America and 10 in Europe) with study populations ranging from 50,000 to 9 million people. Most studies used general practitioner consultations or visits to hospitals for acute gastroenteritis (AGE) as health outcomes. In 11 of the 17 sites, a significant and plausible association was found between turbidity (or particle count) in finished water and the AGE indicator. When provided and significant, the interquartile excess of relative risk estimates ranged from 3–13%. When examined, water temperature, river flow, and produced flow were strongly associated with the AGE indicator. The potential of TSS for the study of the health risk from fecal pathogens in tap water is limited by the lack of specificity of turbidity and its site-sensitive value as an exposure proxy. Nevertheless, at the DWS level, TSS could help water operators to identify operational conditions most at risk, almost if considering other water operation indicators, in addition to turbidity, as possible relevant proxies for exposure.

## 1. Introduction

In 1992, the Milwaukee Cryptosporidiosis outbreak revealed that sophisticated urban drinking water treatment plants (DWTP) did not always fully prevent fecal contamination of finished water, resulting in disease outbreaks. This event also raised the issue of the endemic share of waterborne infections (sporadic cases of disease) in urban facilities [1]. To date, results from the few randomized trials [2,3,4] and case-control intervention studies [5,6] investigating this issue have provided inconsistent risk estimates, leading to controversy about the possible presence of methodological flaws resulting in an inherent bias in risk estimates [7,8]. These studies may also have been too short to prevent the evaluation of risk estimates being overly weighted by specific one-off situations [9]. Insufficient study duration and sampling frequency are primarily due to the cost of water microbial analyses.

In the 1990s, the development of healthcare databases provided new resources for public health surveillance. Concurrently, continuous monitoring of water turbidity and chlorine became more widespread, making large datasets available which could be used to build proxies for exposure. The availability of both health and water records encouraged the use of time series studies (TSS) [10]. Since data were continuously available and free, TSS could last long enough to meet the study requirements in terms of adequate statistical power and representativeness for the most common combinations of epidemiological and hydrological contexts, and yield a robust estimate of the risk.

Turbidity measures the light diffraction capacity of suspended particles in water to estimate the particle load. By analogy with a study of the adverse effect of air pollution on health [11], where the measurement of fine particle concentration in air was extensively used as an exposure surrogate, epidemiologists believed finished water turbidity could act as a possible generic proxy for the pathogen load in water.

In this paper I reviewed published and unpublished water TSS. A quite similar review, carried out by Mann et al. in 2007 [12], covered nine studies. A second one, released in 2017 by De Roos et al. [13], incorporated 14 studies, but it did not include the reports of the multicentric study of the French public health agency [14,15,16,17,18]. Furthermore, the authors of both reviews focused on water turbidity, while water monitoring offers additional daily recorded indicators worthy to be considered as possible complementary proxies for the remaining contamination of finished water by fecal pathogens. In the discussion, I propose a renewed interpretation of operational risk factors and highlight the scientific strengths and limits of the TSS approach from a public health perspective.

## 2. Materials and Methods 

My review process complied with the “Preferred reporting items for Systematic Reviews and Meta-Analysis” (PRISMA) recommendations [19].

I first collected all published TSS addressing the association between water turbidity or precipitation and acute gastroenteritis (AGE). My search of the Medline database (the last search was in November 2017) was based on the occurrence of (“Time series*” AND (“Drinking water” OR Waterborne) AND (Gastrointestinal* OR gastroenteritis OR Diarrhea OR Infection) AND (Turbidity OR Precipitation) NOT Review) in the tittle, abstract or keyword list. All terms were MeSH terms, apart from “Time series”, “Turbidity”, and “Precipitation”. No constraint on the publication date was imposed. I completed the search by examining the reference lists in the primary set of articles. I also considered reports available on university and public health agency websites. 

I excluded reviews and original studies according to the following criteria:

- Studies focusing on an outbreak;
- Studies which design prevents the marginal risk estimate;
- Prospective studies which used self-reported health outcome were excluded to prevent reporting biases due to individuals’ perception of exposure. Only health care-related databases were used, including on-line remote diagnosis data from calls for medical advice;
- Studies showing inadequate mathematical control of potential confounding factors (i.e., resulting in bias);
- Studies where the number of AGE cases included was under 1000, (excluded to prevent insufficient statistical power);
- Studies with a duration of two years or less (excluded to achieve minimal representativeness of the hydro-epidemiological conditions diversity); and
- Studies mapping complex distribution zones (DZ) resulting in major exposure misclassification.

From the selected articles and reports, I first characterized the studied population and the health outcomes. I then characterized the type of water resources, treatment, fecal contamination of raw water (fecal bacteria indicators were under the limits of detection in all sites’ finished water), and the turbidity or particle load in raw and finished water. When the authors did not specify the treatment or the quality of the raw water, I recovered further information from the internet. 

I then focused on the risk associated with turbidity in finished water (Tu_FW), in raw water (Tu_RW) or precipitation. I considered associations which met the following conditions:

- Minimal significance (*p* < 0.1) of the turbidity-risk function (TRF);
- Sufficient plausibility of the shape of the TRF: an increasing TRF was required for the shape to be considered plausible; and
- Good fit between (1) the turbidity-AGE latency observed in the model and (2) the delay for both water transit through the distribution network and infection incubation in sick people.

I also evaluated the robustness of the TRF through documenting (i) the consistency of the TRF over several consecutive lags; (ii) its reproducibility in different age classes, in different health indicators, and in mono- and multi-exposure models (i.e., where several exposure proxies were included together in the risk model); and (iii) when extreme values of exposure were excluded from the dataset.

When possible, I homogenized the exposure scenarios used by the authors to express risks, in order to enable comparisons of the risk levels between the studies. To that end, I expressed the excess of relative risk as both a conventional increase in turbidity of finished water (e.g., 0.01 Nephelometric Turbidity Units (NTU)) and an inter-percentile increase (e.g., inter-quartile (IQ) change). The latter method enabled me to compare inter-site risks irrespective of turbidity levels. When the TRF levelled off at high turbidity values, the P10–P50 scenario (turbidity change from 10% percentile to median) was used instead of the IQ scenario.

Finally, I synthesized the exploratory approach performed in some studies (covering nine French sites), which consist in drawing additional exposure proxies from water operation data to complement turbidity.

## 3. Results

### 3.1. Selected Studies

I defined a drinking water system (DWS) as a distribution network and the resources and the drinking water treatment plants (DWTP) which feed that distribution network. “Site” refers to the urban area serviced by one or several DWS.

**Figure 1:**
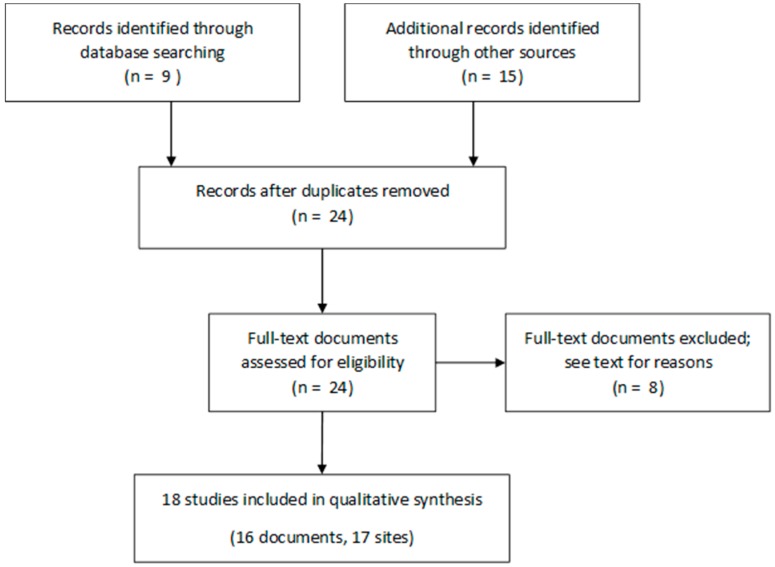
Preferred reporting items for Systematic Reviews and Meta-Analysis (PRISMA) flow diagram of the selection of the studies included in the review.

From the reference search request, I identified nine articles (Figure 1). Including other articles and reports quoted in these nine articles, and reports posted on the French National Public Health agency’s website, I finally gathered 24 documents (17 articles and seven reports) concerning 22 different sites (20 urban, two rural). One report included three different sites studied separately [17] and, therefore, considered separate studies here. I did not distinguish between different DWS which were fed by similar resources and subject to pooled analyses.

From these 26 studies (24 documents) I excluded:

- The Milwaukee studies [1,20,21] which specifically addressed the Milwaukee outbreak and the period preceding the outbreak where other possible contamination episodes by *Cryptospodidium* oocysts may have occurred;
- The Egorov et al. study [22] because of its short duration, the small population size, and the use of self-reported health outcomes;
- The Drayna et al. study [23] because it focused on the relationship between precipitation and AGE in Wisconsin (USA), irrespective of distribution zone (DZ) locations and organization;
- The Harper et al. [24] and Uejio et al. [25] studies focusing on rural areas; and
- The first study carried out in Le Havre [26] that used a moving average model yielding conditional risk estimates.

Among selected sites, several DWS shew complex production-distribution patterns with DZ fed by several DWTP (Le Havre, Paris, New York) and including sectors with time-varying water origin; but no exposure misclassification remained at the population level, since adequate operation data (e.g., daily records of turbidity and produced flow) were available for all DWTP involved, thus allowing to calculate unbiased exposure estimates (e.g., mean turbidity weighed by produced flows).

Eighteen studies, therefore, met selection criteria (Table 1), and concerned 17 different sites corresponding to nine articles and seven reports. Seven sites were located in North America, nine in France, and one in Sweden. Among the American sites, three covered several DWS, but with DWTP servicing separated DZ (no risk of misclassification): Philadelphia, Vancouver, and Atlanta.

**Figure 2:**
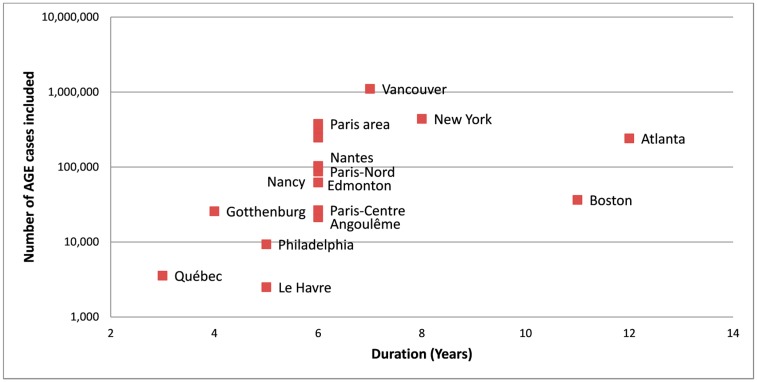
Duration of the selected studies and number of acute gastroenteritis (AGE) cases included.

With respect to the statistical power of the selected TSS and the robustness of the risk estimates (Figure 2), four studies included fewer than 10,000 AGE cases: the study performed in Le Havre, the two in Philadelphia, and the study in Québec. The second Philadelphia study lasted only two years and its study periods was embedded within the period of the previous study, while the Québec study lasted three years. In contrast, I counted 10 studies each with a study period of at least six years and each with approximately 100,000 AGE cases: Atlanta (with the longest study period of 12 years); New York; Nantes; Paris-Est; and the three sites in the Paris area (PA), defined as follows: PA-Nord, PA-Est (N.B.: not to be confused with Paris-Est), PA-Sud, Nancy, Vancouver (with the largest dataset of 1.1 million included cases), and Boston.

### 3.2. Serviced Populations and Water Systems

Table 1 summarizes all the characteristics of the selected studies and sites and provides references. The size of the studied populations was greater in North America than in other countries: five of the seven sites there provided over one million people with tap water (maximum, New York: 9.2 M; minimum, Québec: 240,000). In contrast, only one of the 10 European sites included supplied over one million people (maximum, PA-Sud: 1.4 M; minimum, Angoulême: 50,000).

I distinguished three types of DWS according to the type of resource: (1) “river DWS” fed by rivers with moderate to high fecal pollution and which had treatment systems incorporating full clarification facilities, i.e., at least coagulation-flocculation-decantation and rapid sand filtration facilities; (2) “reservoir DWS” served by protected reservoirs and treatment facilities limited to disinfection; and (3) “karst DWS” fed by karstic springs and involving diversified treatment processes including clarification or not.

Eleven river sites were fed exclusively by river DWS. This category was relatively homogenous in terms of both resources and treatments. Eight were fed by a single DWS: Nantes, Québec, Paris-Est, Gothenburg, PA-Nord, PA-Est and PA-Sud, and Nancy. The remaining three were fed by several DWS: Philadelphia, Edmonton, and Atlanta. Atlanta exhibited the more complex scheme with three resources, nine DWTP, and eight DZ. The potential heterogeneity in exposure in the serviced populations in these three sites was limited by the sharing of common resources and treatments. Mean turbidity and *E.coli* concentrations in resources ranged from moderate (<5 NTU and <100 FCU/100 mL) in Gothenburg and Québec, to high in the Paris-Est and the three PA sites (>15 NTU and >1000 FCU/100 mL). Both particulate and fecal contaminations were higher following heavy rain episodes, in proportion to each site’s baseline contamination level (40–100 NTU and 500–50,000 FCU/100 mL). All 11 river DWS had full clarification treatments, including, at least, coagulation-flocculation-decantation (CFD) facilities. Paris-Est additionally operated slow sand filtration downstream of CFD. The PA-Nord site used membrane ultra-filtration in one of the two plants servicing the DZ. DWTP operated ozonation in seven of the river sites. In the other four sites, chlorination (Philadelphia, Edmonton) or UV irradiation (in some DWTP feeding Atlanta and PA-Nord) were operated, followed by post-chlorination. Given the high efficiency of clarification facilities, Tu_FW was kept very low (mean <0.07 NTU), except in two American sites: Philadelphia (0.17 NTU), Québec (0.27 NTU). *E. coli* was not detected in any of the 11 river DWS.

The “reservoir sites” category comprised only three North American sites: Vancouver, divided in three reservoir DWS, Boston, and New York, which were fed by a single DSW. This category was also relatively homogenous in both resources and treatments, being limited to disinfection, and consequently finished water qualities were also similar. Due to wildlife protection policies in the USA and Canada, mean *E. coli* concentrations were low (1–2 FCU/100 mL) but could rise slightly following heavy rain episodes or when geese were present on the water surface; *E. coli* maxima ranged from 14 to 57 FCU/100 mL depending on the resource. In addition, baseline turbidity levels were low to moderate (0.3 NTU in Boston and 1.3 NTU in New York and Vancouver) and rose when algal blooms or muddy runoffs occurred, with daily maxima staying below 1 NTU in Boston and 3 NTU in New York, but reaching 19 NTU in Vancouver reservoirs. In the absence of clarification facilities, turbidity did not significantly change from reservoir to taps. Again, *E. coli* was not detected at any time in finished water thanks to heavy chlorination (Vancouver, New-York) or ozonation (Boston).

Karst water may be viewed as groundwater episodically mixed with surface water from a flooding river or surface runoff. Baseline microbial contamination and particle load vary substantially between these two hydrological stages (recession in dry weather vs. turbid high water following heavy rain episodes), as well as between springs. The “karst sites” category comprised three French sites, each corresponding to one DWS: Le Havre, which was fed by two different springs and DWTP, referred to as systems 1 and 2, Angoulême and Paris-Centre. The hydrology of the Paris-Centre and Le Havre (syst. 2) aquifers showed discrete karstic patterns, with turbidity varying from 0.1 NTU in dry weather conditions to 0.5–1 NTU in wet weather conditions and fecal contamination from 1 to 14 FCU/100 mL. On the contrary, Le Havre springs (syst. 1) had higher baseline contamination (4 NTU and 80 FCU/100 mL) and were hit by frequent and intense periods of high turbidity (>100 NTU and >1000 FCU/100 mL). Tu_FW depended on both Tu_RW and the clarification process: in sites with no clarification facilities, i.e., Paris-Centre and Le Havre (syst. 2), turbidity did not change from the springs to the tap, while in Angoulême, coagulation and rapid sand filtration achieved the local target of <0.2 NTU in finished water. In the Le Havre DWTP (syst. 1), water was only filtered in dry weather and decantation was implemented when turbidity in the spring water exceeded 3 NTU. Consequently, consumers experienced lower Tu_FW in wet weather conditions (0.1 NTU) and higher Tu_FW in dry weather (0.4 NTU). *E. coli* was not detectable at any time in finished water from karst DWS.

### 3.3. Health Outcomes

AGE is used in epidemiology as a generic indicator for infections arising from fecal pathogens, as the syndrome is very common [40,41]. This provides potential sensitivity, and as AGE has a short incubation period, it also provides good reactivity to environmental triggers. AGE indicators used in TSS depend on the availability of syndromic data, i.e., on the local health care system. Indicators should, however, meet sensitivity and specificity if referring to a symptomatic definition of a case to achieve an accurate risk assessment, and remain stable over time.

In the US, the availability constraint led epidemiologists to examine visits and admissions to hospital emergency departments from different data providers. Both diagnosis by medical staff and the use the International Classification of Diseases (AGE related codes in ICD9: 001 to 009.9; 276; 558.9; 787) for coding, guarantee a high and stable specificity of the health indicator. Furthermore, sensitivity towards the symptomatic definition of AGE cases is low as the cases admitted to, or visiting, the hospital are the most serious. In the USA, with all routes of infection and causal pathogens being taken into account, the incidence rate of visits to emergency hospital departments was five visits a year per 1000 people in the Atlanta study, whereas 17 visits a year per 1000 people >65 years old was measured in hospitals in Boston, resulting in two elderly patient visits per 1000 people a year in the general population. The sensitivity may vary across time according to the capacity of the patients to pay their health care bills. That is one of the reasons why some authors [27,34] restricted their recruitment to elderly people supported by the Medicare program.

In France, epidemiologists used prescriptions of General Practitioners (GP) from a health insurance database [42]. The indicator results from the application of an algorithm based on the test of the nature and quantity of prescribed drugs, which enables true cases of AGE to be discriminated from others. The evaluation of this algorithm [42] concluded that sensitivity was acceptable (approximately ten percent of true medicalized cases were wrongly rejected) and specificity (approximately ten percent of included cases were not cases of AGE). The indicator was also calibrated with the consensus symptom-based definition of AGE cases [43]: MG consultations accounted for 33% of total cases, according to Majovicz’s definition [41]. Thus, the French health insurance database provided enough sensitivity to perform separate analyses in children and adults in cities with over 50,000 people. According to a case definition based on drug prescription analysis with all routes of infection being taken into account [42], the mean incidence rate of GP consultations for AGE in France was approximately 47 a year per 1000 people in Nantes and 90 in children under 16 years of age, resulting in 14 pediatric cases a year per 1000 people, when taking the whole community as the denominator. Incidence rates of AGE prescription were quite similar in other French cities. Again, the prescription-based indicator was moderately insensitive to the variations of patient’s income, because health expenditures are largely covered by the French social security system. Furthermore, the reduced turnover of drugs dedicated to AGE care, and the yearly adaptation of the discriminating algorithm to changes in medication, made it possible to maintain a relatively constant specificity of the indicator over time.

Other data sources were used to perform TSS. Prescribed and over-the-counter drug sales were used in the Le Havre study, and phone calls for medical advice in the Québec study (with a yearly incidence rate of 19 per 1000 people). In France, drug sales are a very sensitive and relatively stable indicator of AGE case, but specificity is poor [42]. I did not find in the literature a systematic evaluation of the phone call data for epidemiological use.

### 3.4. Model Designs

In all the TSS included in the review, authors basically used Poisson regression most often adapted to over-dispersed counts (e.g., by using quasi-Poisson regression) to model daily counts of AGE cases and implemented the generalized additive model (GAM). When specified, the criterion for fitting the model was the absence of autocorrelation in residuals, which provides optimal risk estimates when health outcomes follow marked seasonal variations [44,45]. Control covariates were mostly similar: trends and seasonal patterns were modelled with a spline or Loess function of time, day of the week, and school vacation periods.

The main differences between the studies reviewed stem from the different sets of exposure variables included in the models used (Table 2), from the shape of the risk functions, and from the width and position of the time window used to average exposure variables included in the GAM. Some authors forced the linearity of the TRF, whereas others considered non-linear risk functions modelled by spline or Loess functions. I also observed a large diversity in the width of the time window used for exposure assessment. The earliest studies used single day windows over 0–15 or 0–40 day lags [33], whereas later studies calculated means over several consecutive lags (e.g., 5–7 up to 0–21). All French studies systematically assigned a three-day width to the time window to take into account the spread of response and a six- or seven-day delay for the start of the window, in order to exclude the latency period. One study [32] used a distributed lag non-linear model over a 0–21 day lag to optimize the time window shape instead of forcing it to be rectangular.

### 3.5. Turbidity-Related Risk

Significant, robust and plausible associations between Tu_FW and AGE incidence were found in 9 of the 16 sites where Tu_FW was tested as a proxy of exposure (9 positive/16 total sites tested, since Gothenburg did not test turbidity, but only precipitation, as a candidate exposure variable). Moreover, in Atlanta, the risk associated with Tu_FW and estimated using pooled data from all DWS was noticeable, but not significant, and the risk latency was consistent with the expected latency (see Discussion: Standardizing the Time Window for Exposure Assessment). The insufficient resolution of the turbidimeters at very low turbidity levels (Tu_FW < 0.05 NTU) may have prevented the authors from observing a possible risk associated with the presence of suspended particles in finished water in the Paris area sites, but the availability of particle count data (particle size detection range 1.5–15 µm) for PA-Nord and PA-Sud enabled them to do so. Considering both turbidity and particle counts, a significant association between the particle load of finished water and AGE was observed in 11 of these 16 sites (11/16). I did not observe significant differences in the frequencies of positive results between the three DWS categories, but all DWTP servicing people with unfiltered water exhibited a significant and plausible turbidity related risk (5/5). Tu_RW was also correlated to AGE (five positive in 10 sites equipped with clarification facilities). In two of these four positive sites (Atlanta, Angoulême) Tu_FW was not significantly correlated to AGE. Precipitation, which can be viewed as the primary driver of turbidity, was rarely correlated to AGE in the reviewed studies (1/10). Daily measurements of *E. coli* in resource water were rarely available and, consequently *E. coli* was rarely tested as a possible indicator of exposure. It did not closely correlate to AGE incidence (1/3).

**Figure 3:**
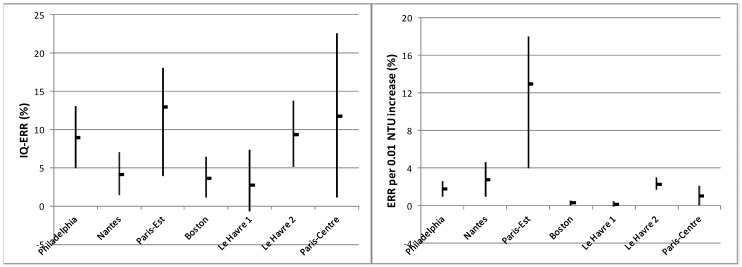
Excess of relative risk (ERR) associated to turbidity increase in finished water in six urban areas.

As health risk was modelled as a function of the turbidity, in order to express the size of the risk using only one number, an exposure scenario needs to be chosen. Two types of scenarios were used: an absolute increase in turbidity and an interquartile variation in turbidity. The first leads to an expression of the risk which is dependent on turbidity, whereas the second leads an expression of the risk which is site-dependent. I was able to calculate both expressions for six DWS corresponding to seven DWTP (Figure 3). The average interquartile (IQ) excess of relative risk (ERR) ranged from 3–13%, whereas the averaged ERR per +0.01 NTU was between 0.2% and 13%. ERR were generally higher in children [14,15,17,31] and in the elderly [27,33] than in adults. In Philadelphia, elderly people >75 years old were at a higher risk than those aged 65–74 [27]. In the five French studies where medical prescriptions were used as health outcome, the children-adult ERR ratio was between one and three. In the New York study, authors found similar ERR levels in both children and the population as a whole, and did not notice any turbidity-AGE association in the elderly.

In the Vancouver study, authors systematically tested both hospital admission and visits to GPs and found that hospital admission data yielded a risk attributable to tap water turbidity which was approximately half that from visits to GPs (0.6% vs. 1.6%).

The shapes of the risk function were not always linear or quasi-linear. On the contrary, the use of spline functions showed that non-linearity was a common feature. In four sites (Nantes, Boston, Paris-Centre, and Paris-Sud), non-linear TRF leveled off and became inaccurate at high turbidity values. This suggests that an additional variable was needed in the model to correctly characterize the risk at high turbidity.

### 3.6. In Search for Additional Exposure Proxies from Water Operation Data

**Figure 4:**
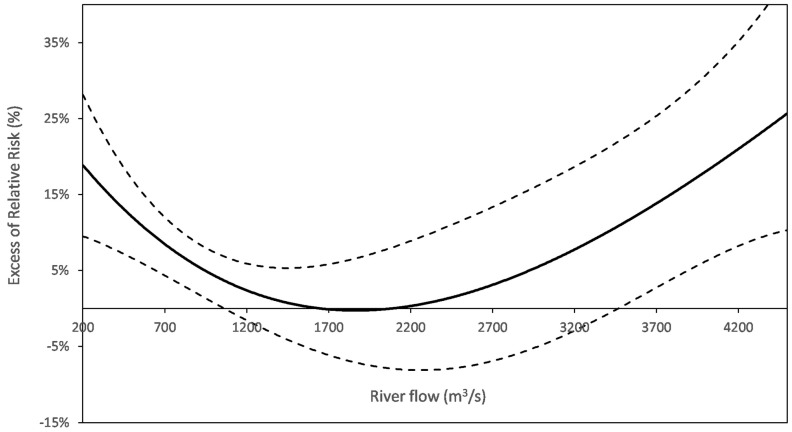
AGE risk function for the river flow (Nantes). The right branch may miss (e.g., PA sites). The delay considered for the health response to the river flow is homogenous with that used for turbidity.

**Figure 5:**
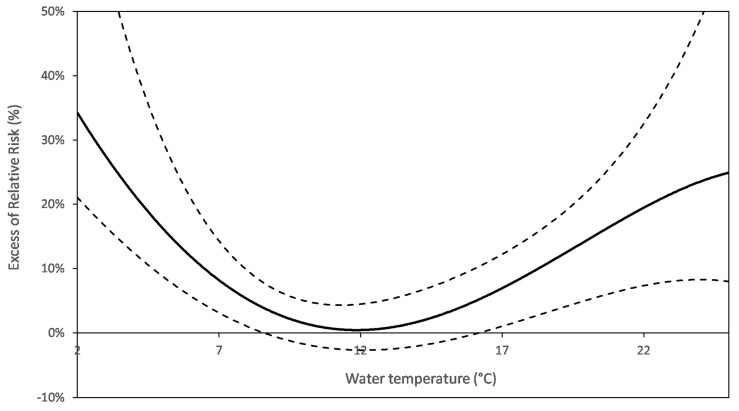
AGE risk function for the water temperature (PA-Nord). The right branch may miss (e.g., Boston). The delay considered for the health response to the river flow is homogenous with that used for turbidity.

**Figure 6:**
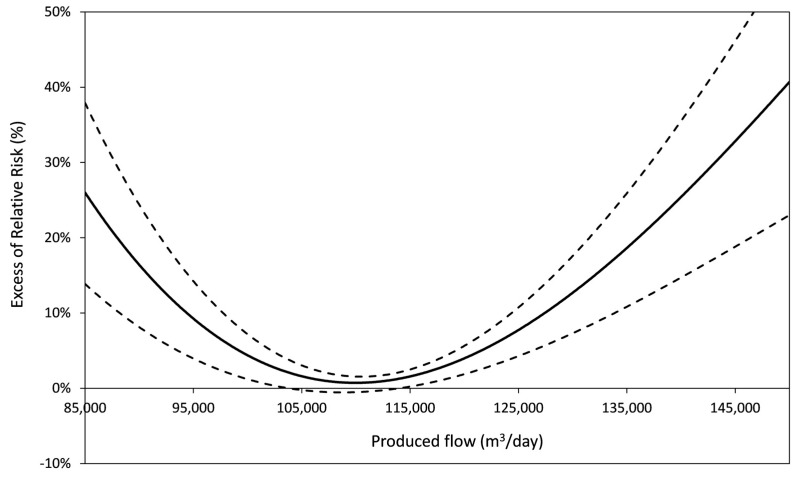
AGE risk function for the produced flow (Nantes). The left branch may miss (e.g., PA and Paris sites). The delay considered for the health response to the river flow is homogenous with that used for turbidity.

In all of the French studies, water operation and weather data were systematically explored to identify alternative or complementary proxies of exposure to waterborne pathogens. Fourteen variables were tested in total, or 22 when distinguishing the different measurement points inside the treatment chain (e.g., Tu_RW and Tu_FW) and derived variables (e.g., the number of consecutive dry days derived from the precipitation time series) (Table 2). Some variables were tested only once and others repeatedly, according to the availability of data. This exploration stage highlighted four variables as major contributors to the risk model: qualitative change in treatment (2/2), river flow for river DWS (5/6; Figure 4), supply water temperature (6/6; Figure 5) and produced flow (8/9; Figure 6). The levels of risk associated with these alternative exposure proxies were higher and more significant than the risk associated with turbidity. Since no strong collinearity was observed within the exposure set or between exposure levels and control covariates, risk estimates did not change with the removal of other exposure variables from the model. In addition, variations in the dataset used to adjust the model, e.g., adult instead of children health data, did not affect the set of significant exposures or the shape of the risk functions.

The Le Havre study showed that decantation—triggered when Tu_RW exceeded 3 NTU (syst. 1)—strongly modified the TRF associated with Tu_FW, with a shift from a high risk (IQ-ERR = 18% (14; 22)) in the absence of decantation, to a low and non-significant risk (IQ-ERR = 3% (0; 7)) with decantation. As well, the Boston study highlighted a possible decrease in risk due to the change from chlorination to ozonation. However, the disinfection-related proxies were generally not correlated to AGE incidence, except in one case (1/4).

River flow was a consistent factor in exposure (5/6). Basically, the U-shaped relationship between river flow and AGE indicated an increased risk at extreme flows (Figure 4). Both branches of the U could co-exist (e.g., in Nantes), whereas only the falling left branch remained in Paris-Est and the three sites in the Paris area, exhibiting a risk at lowest flow. Water temperature (6/6) or air temperature, as surrogates of water temperature (3/4), were also factors in exposure (6/6), with an associated risk exceeding by far (Boston) the turbidity related risk. As with the river flow, the temperature-risk functions decreased (Boston, Paris-Sud) or were U-shaped (other French DWSs), indicating that the risk was concentrated at extreme temperatures.

River flow also modified the TRF in Nantes and Paris-Est, and the water temperature in Boston and PA-Sud. These interactions (i.e., river flow—turbidity and water temperature—turbidity) were modelled as a tensor product smooth (TPS) which provided better estimations of risk than the cubic splines of each variable, taken together. The examination of the bivariate relative risk TPS (Tu_FW, river flow) and TPS (Tu_FW, water temperature) provides some clues to help explain the common lack of accuracy of the TRF at the high-level domain of turbidity (e.g., Nantes, Paris-Est, and Boston). Higher risks resulted from a combination of high Tu FW and extreme river flow (Nantes) or only low river flow (Paris-Est). In Boston, low temperature combined with turbidity, increased the risk whereas, in Paris-Est and PA-Sud, high temperature combined with Tu_FW or particle counts, respectively, increased the risk. Season may also modify the TRF, as was the case in New York, where AGE incidence was only related to drinking water turbidity after the spring snowmelt.

Produced flow was correlated to AGE incidence (8/9). The risk function varied from linearly increasing to U-shaped (Figure 5). The authors did not observe any interaction between the produced flow and other exposure proxies. IQ-ERR (when the risk function was increasing) or P50-P90 ERR (U-shaped risk function) often exceeded the corresponding turbidity related risk estimates.

No proxy related to distribution incidents (e.g., leaking pipes, cuts in the water supply) or repair interventions was strongly associated with the AGE incidence (overall: 2/5, details in Table 2).

Considering the subset of multi-exposure models, I calculated, when available, the difference in risk between all the exposure variables (Tu_FW or particle count, water temperature, produced flow, and river flow for river DWS) at the 75th percentile (in the direction of increasing risk) and at the 25th percentile. I observed, in most cases (4/6), that the contribution of Tu_FW in the IQ-ERR sum was lower than that of two or three other variables (max = 33 %).

## 4. Discussion

### 4.1. An Underestimated Established Risk

“It is likely that an association between turbidity and gastro-intestinal illness exists in some settings or over a certain range of turbidity”, concluded Mann et al. in their review [12]. The findings of the updated review by De Roos et al. (12 sites studied vs. five in Mann’s review) confirm that conclusion, as well as this review, which has kept nine sites in common with the De roos’ review and incorporated seven additional sites. With 11 sites exhibiting an association between particle content of finished water and AGE incidence, the results from TSS clearly favor the existence of a residual risk of AGE from tap-water intake in the cities of developed countries. Risk could be higher with unfiltered water drawn from reservoirs and karst aquifers. On the other hand, TSS also show that even enhanced treatment (i.e., slow sand filtration instead of rapid filtration in Paris-Est) cannot fully cope with river water of poor microbial quality, and that joint watershed protection and water disinfection measures do not lead to an undetectable risk in people serviced by reservoir DWS.

The design of TSS is suitable for studying risks which vary from day to day. TSS are, however, unable to capture the full risk arising from fecal contamination of tap water. Specifically, TSS cannot capture an (improbable) time-steady risk, nor the risk acquired during the distribution stage, e.g., backflows of contaminated waters into the distribution network which cause a significant proportion of waterborne outbreaks [46,47] and possibly sporadic cases [48]. Furthermore, French TSS, which add other exposure proxies to the risk model, show that turbidity does not cover the full risk generated upstream of the DWTP outlet.

### 4.2. How to Improve Inter-Site Comparison of Turbidity-Related Risks

Despite the statistical modelling framework and options being quite similar in almost all the studies included, the diversity of the indicators used in the studies for both health and exposure hindered me from generating a comparison of the risks.

#### 4.2.1. About Health Outcomes

The AGE indicators used in studies depended on their availability, i.e., on the organization of each country’s health care system. Visits and admissions to hospital in North America and GP prescriptions in France are the two main sources of AGE indicators used in published TSS. They demonstrate adequate specificity and stability over time to target symptomatic cases of AGE, thanks to standardized coding (ICD) and regular revision of the discrimination algorithm, respectively. The diversity of AGE indicators corresponded to different degrees of symptom seriousness and to different age groups. Accordingly, the sensitivity of the available AGE indicators dictated the minimum population size needed to obtain sufficient statistical power to establish the presence of a risk. Indicators based on GP activity data were almost 10 times more sensitive than those based on hospital activity, this resulting in different ranges of population sizes between the North American and French sites. Irrespective of the sensitivity level, the stability, (i.e., the limited change in sensitivity and specificity over time) of most AGE indicators used for TSS meets the conditions to carry out TSS.

Children and elderly people were often targeted in the studies reviewed, because (i) they are at higher risk of infection than adults [40,41], and (ii) they are less subject to misclassification of exposure [31]. Indeed, compared with the whole community, these two subpopulations include fewer people who commute daily for work reasons, and so who drink tap water from different DSW. As such, a misclassification is a priori independent of health outcomes, the resulting bias is not differential, and only brings the risk estimate to zero. In the French studies, where models developed in children were systematically tested in adults, risk levels in adults were approximately half those in children. Both higher susceptibility to infections and lower misclassification rates could have participated in generating differences in estimated risk levels. The choice to study one particular population (i.e., children or the elderly), as opposed to studying the population as a whole, results in a trade-off between the loss of statistical power due to population restrictions and underestimation of the risk attributable to misclassification bias, arising from people commuting to areas with different DWS.

#### 4.2.2. Need to Standardize the Time Window for Exposure Assessment

One practical condition needed to make an accurate inter-site comparison of the risks of gastroenteritis in tap water is that the formulation of the turbidity-based exposure variables must be similar between studies. In this review, the diversity of the time windows used for exposure assessment—from the earlier studies which used daily mean turbidity as exposure to the Atlanta study where a 0–21 day window was considered for statistical testing—advocates for future standardization. Exposure time windows should exclude the latency period accounting for water transit to consumers’ taps (e.g., 1–2 days), incubation of AGE causing pathogens (e.g., 1–10 days and 4–6 days for the modal delay) and search for medical help (e.g., one day). The width of the window should also match the distribution of the incubation durations of all pathogens potentially involved in the risk, and the variability in the times of water transit from the drinking water treatment plant (DWTP) outlet to taps. Restricting the time windows (e.g., 6–10 days) to exclude inconstant, if not implausible, early and late responses would facilitate the comparison of risks between studies.

#### 4.2.3. Need for Long Duration Studies

In addition to providing increased statistical power, a longer study duration would most probably have a crucial effect on the robustness of the risk estimates, because the contamination of finished water results from highly diversified combinations of pathogen shedding, resource contamination and treatment options to be covered by the study period.

Basically, hydrological conditions drive the fecal contamination of surface resources and of aquifers influenced by surface waters. In dry weather, fecal contamination is minimal, with a baseline level depending mainly on human pressure, i.e., the discharge of upstream waste water treatment plants (WWTP) located upstream of the DWTP intake. In the case of urban rivers, at the lowest flows, discharges from WWTP are less diluted and may cause a dramatic increase in the fecal contamination of river water, e.g., in the Paris area sites. Rain downpours first result in urban runoff and sewage overflow [49] causing the release of raw urban waste water (i.e., fecal pollution of human origin) into the rivers. Stronger or extended episodes of rain may further trigger rural runoffs contaminated by cattle and wildlife or manure spread on fields. In addition, sudden rises in river flows cause the re-suspension of the contaminated mud previously settled on river beds. Accordingly, high flows most often correspond to high fecal contaminations (e.g., 2 log-units or more above baseline) of surface water and of influenced groundwater bodies.

Fecal pollution in raw water does not necessarily mean a high pathogen content. The shedding of a given pathogen may vary yearly, seasonally, and in much shorter timescales, depending on the pathogen carriage level in humans and animals. Consequently, at a given time, fecal indicators may be correlated to the presence of one dominant pathogen (e.g., norovirus in January), several pathogens (as some outbreak investigations have shown), but may also occur in the quasi-absence of pathogens in resources, as shown by the fact that fecal accidental contaminations of drinking water outnumber outbreaks.

Finally, in addition to resource contamination by pathogens as a necessary condition, the contamination of finished water also necessitates that pathogens break through barriers provided by treatment. For sophisticated DWTP, permanently maintaining the barrier effect of treatment challenges the capacity of timely treatment adaptation to short-term changes in raw water quality, especially peaks in turbidity and organic matter.

To summarize, finished water contamination involves three time-varying conditions (shedding of pathogens, transport, and inadequate treatment) combining in a large variety of events. Thus, robust risk assessment not only requires the inclusion of a sufficient number of AGE cases but also long duration study periods, e.g., 5–10 years (Figure 2). Accordingly, the findings of long-duration studies (Atlanta, Boston) are more reliable than short duration studies (Québec, Gothenburg) where the risk functions may be attached to one-off situational conditions.

### 4.3. Microbiological vs. Operational Interpretations of Turbidity

“Microorganisms are only a tiny portion of the total number of suspended particles in water and pathogenic microbes are likely to be only a tiny fraction of the total microbial population” [27]. Given this, turbidity cannot be simply considered an indicator of pathogen content, but only a surrogate, the relevance of which relies on the statistical association between turbidity and pathogen load, i.e., the rain-driven concurrent presence in water resources of particles from soil erosion and pathogens from sewage overflow and manure entrainment.

The association between organo-mineral particles and pathogens is not only statistical, but also physical: bacteria may form biofilms on particles [50]. Furthermore, other viral and protozoan pathogens may also bind to particles [51,52]. Some experiments have shown that most bacteria, viruses, and protozoa in treated waste waters, as well as surface runoffs [52] and natural waters [53,54,55], are attached to, or embedded in, organo-mineral particles suspended within the water body. Accordingly, organo-mineral particles may act as Trojan horses, permitting the entry of infectious pathogens into distribution networks.

Chemical disinfection is expected to ensure that particle-free fecal bacteria are totally inactivated, and free viruses are totally or partially inactivated, depending on the disinfectant and dose used. However, it may fail to inactivate protozoan parasites, especially *Cryptosporidium* [56], although the World Health Organization stated that high rates of ozonation can bring about *Cryptosporidium* inactivation [57]. Studies performed in real conditions show that pathogen inactivation rates vary substantially in time and may be episodically low [56]. Some factors of ineffective disinfection have been well described, for example low temperatures or dead zones in disinfection reactors. However, the key factor when focusing on turbidity as a proxy for exposure is that suspended particles in water greatly hinder disinfection by harboring pathogens [57,58]. This is an especially important issue for waters from reservoirs and karsts which are distributed without clarification. However, information in the literature on the particles-pathogen association and the harboring effect which protects against disinfection is scarce.

Additionally, little is known about the relationship between particles and pathogens in clarified waters [59]. The key condition to efficient rapid sand filtration, i.e., which significantly retains pathogens, is upstream well-operated coagulation [57]. Hijnen and Medema recommend conservative values for real-condition removal rates of coagulation-filtration: 0–4 log for viruses, 1–3 log for bacteria and 1–5 log for *Cryptosporidium* oocysts [56]. Discrepancies between operational and theoretical removal rates, the latter corresponding to the upper boundaries of the aforementioned ranges, reflect difficulties in maintaining the efficiency of treatments all of the time when faced with changes in raw water quality.

Although some biological elements of plausibility advocate for the use of turbidity as an indicator for exposure to pathogens, the findings from this review of TSS do not entirely support this. The expression of ERR by 0.01 NTU led to over-dispersed estimates of risks (0.2–13%) among the six different DWS sites where ERR was calculable (Figure 3), whereas the range of estimated IQ-ERR values was tighter (3–13%). Moreover, ERR, whatever their expression, were independent of Tu_FW baseline levels. These observations question the biological interpretation of Tu_FW as a universal proxy for the pathogen content of finished water. On the contrary, they suggest that Tu_FW could be considered a site-specific proxy for the general functioning of a DWTP and that only relative changes in Tu_FW matter in risk assessment. This latter interpretation also rejects the impossibility of observing a significant risk below 0.2 NTU, which was argued in the earliest TSS [7].

In conclusion, there are two possible and, in a certain extent, compatible interpretations of turbidity as a proxy for pathogen exposure: the biological interpretation and the operational interpretation. The operational interpretation may be more suitable for river DWS, as Tu_FW mainly reflects the accuracy and timeliness of on-line treatment adaptation to changes in raw water quality, whereas turbidity in unfiltered reservoir water can be more directly interpreted in terms of environmental conditions.

### 4.4. Public Health Issues

#### 4.4.1. TSS Do Not Support the Quantitative Health Impact Assessment

In a former review [60], authors stated that the set of published TSS is still inadequate to draw a meta-risk estimate representative of the risk in cities of developed countries. The insufficiency of the dataset not only results from the small number of available studies and from the recruitment provisions leading to an overrepresentation of “good performers”, but mainly from the intrinsic heterogeneity of turbidity that makes pooled analyses irrelevant.

The quantitative health impact assessment [61] aims to predict the possible effect of prevention actions on risk. It is based on the existence of a robust causal risk function. TSS do not offer an interesting perspective for the building of a generic meta-TRF. Even when considering a single site, the specific TRF (if any) does not enable operators to forecast the effect on risk of a reduction in turbidity. For instance, the implementation of enhanced treatment facilities (e.g., clarification) probably lowers the turbidity baseline in finished water, but also changes its composition by differential selection of particles, according to their size, shape, density, and electric properties. The relationship between Tu_FW and the pathogen presence would also probably change under new treatment conditions, as some real-world experiences of decantation implementation have suggested [36], making the TRF irrelevant for such inference.

#### 4.4.2. TSS Do Not Help Identify Causative Pathogens

The description of the DWS provides some clues about the pathogens which may survive in finished water. In the case of reservoir DWS, the exclusion of human activities and livestock from the reservoir watershed makes wildlife the main (theoretically, the only) source of pathogens: the pathogens at the inlet of DWTP should, therefore, encompass bacteria and protozoa and exclude viruses. Infective protozoa may only possibly remain at the outlet of DWTP, considering the strength of the disinfection implemented. On the contrary, the finished water from river DWS should mainly contain viruses since humans are probably the main source of pathogen discharge into the river in urbanized watersheds. Protozoa may also be present in the case of inefficient clarification [1]. For all studied DWS, the presence of infective fecal bacteria is improbable because of the disinfection provisions.

To date, results from TSS have added little information about the causative agents involved in water-related AGE cases. Long latency in the health effects associated with the reservoir DWS turbidity, i.e., more than two weeks, could suggest the presence of protozoa in finished water [1,33,34], whereas the early response suggests pathogens with short incubation durations, e.g., viruses. Alternative interpretations of late responses may, however, be put forward: (i) long distribution time in remote parts of the distribution network; (ii) secondary cases infected by contact with primary water-related cases [20]; or (iii) increased incubation times due to lower pathogen doses ingested by consumers than referenced infective doses observed in outbreak investigations or experimented in controlled trials [62].

#### 4.4.3. But TSS Can Teach Water Operators about High Risk Conditions

Risk functions formulated as operation conditions may directly help water operators in developing prevention measures without having to go through a formal pathogen identification step. To improve water microbial quality, multi-exposure models provide more useful information to achieve prevention measures than models where exposure is limited to turbidity. Indeed, this review highlighted three variables worthy of examination as exposure indicators in addition to turbidity: produced flow, river flow (for river DWS), and water temperature.

TSS results consistently (8/9) showed the adverse health effects of the high production of drinking water, irrespective of the effect of turbidity, possibly due to the shortening of transit time of water across treatment facilities and the consequent lowering of retention and inactivation rates. For other DWS, the U-shaped produced flow-AGE functions suggest the existence of optimal flow conditions (Figure 6).

French studies also suggested an adverse effect of extreme river flow on the risk of AGE observed in five of the six sites where river flow was tested. The J- or U-shape of the risk function (Figure 4) is in line with prior knowledge about the drivers of contamination (see § Need for long-duration studies). Furthermore, the modifying effect of river flow on the TRF, highlighted in Nantes and Paris-Est, suggests that, in extreme flow conditions, a similar amount of particles in finished water may shelter (or be associated with) significantly more pathogens than in intermediate flows.

Temperature heavily influences the risk of AGE both as control and exposure (Figure 5). High temperatures enhance the dehydration risk in patients with AGE symptoms and encourage people to seek medical help earlier. The absence of latency in the health effect supports this interpretation. In addition to the likely direct effect of air temperature on symptom seriousness, present in the Boston and New York studies, water temperature (or air temperature as a surrogate) also factors in exposure (9/10), whatever the route (tap water, food, or contact), as suggested by the delayed response of the risk of AGE to temperature, consistently with the incubation duration of the AGE-related infections. Moreover, the presence of interactions between water temperature and Tu_FW on the risk in Boston and PA-Sud advocates for a waterborne effect. Considering the delayed influence, extreme temperatures result in higher and plausible risks. Low temperatures mean greater survival of pathogens in the environment [63,64,65] and slower disinfection [56] and coagulation dynamics [66]. At high temperatures, dissolved organic matter may rise in the surface water and cause an unexpected chlorine demand, thus temporarily compromising disinfection power. In addition to its waterborne effect, water temperature may also be influential in other routes of infection. For instance, cold weather favors indoor confinement and consequent cross-infection from contact between people and fomites. Accordingly, temperature should be involved in waterborne exposure, but only partially.

Some of the identified risk factors are environmental constraints for water operation (river flow, water temperature), while others may only be marginally controlled (produced flow). Turbidity remains the only risk factor that may be lowered by improved operational provisions. However, since decreased turbidity may mean a change in the nature of the particles, cutting the risk by decreasing turbidity could be a false solution when seeking to improve the microbial quality of finished water. Prevention actions could target other factors than turbidity, for instance, increased disinfection during episodes deemed to be at higher risk. Authors should discuss the results and how they can be interpreted in the perspective of previous studies and of the working hypotheses. The findings and their implications should be discussed in the broadest context possible. Future research directions may also be highlighted.

## 5. Conclusions

In the history of the epidemiology of waterborne infections, priority has been given to microbiology to specify both the health effects and the exposure level. When opportunities for an alternative approach appeared, based on syndromic and water operation data, TSS seemed to be a cost-reasonable solution to study waterborne risks in cities.

Despite this advantage, TSS have not caught on in this field for several reasons. Scientifically, the lack of specificity of turbidity as a proxy for exposure appears to be the main limitation to obtaining accurate estimates of the risk at the site level. Furthermore, inter-site heterogeneity of turbidity prevents further meta-risk assessment and limits the review to a qualitative approach. TSS could help provide safer finished water, provided that models increase the set of relevant exposure variables drawn from operational data.

## Figures and Tables

**Table 1:** Description of the selected sites and associated risks.

Site	Period	Serviced Population	AGE Indicator	Age Classes (Years)	Number of Resources, DWTP and DZ Covered by the Study	Type of Resource	Turbidity in Raw Water: Mean (Max) (NTU)	[E.coli] in Raw Water: Mean (Max.) (CFU/100 mL)	Treatment Facilities ^a^	Turbidity in Finished Water: Mean (Max) (NTU)	Number of AGE Cases Included	Exposure Scenario	ERR Related to Exposure Scenario	Significant Lags (Days) ^b^	Proxies Tested as Exposure with Significance and Reproducibility ^c^	Study
Philadelphia (USA)	1989–1993	1.2 M	Visits and admissions to hospital	<16	2-3-3	Rivers	9–19 (100–150) ^d^	20–40 (1000)	PCh, CFD, RFi, pChm	0.17–0.20	3282	Tu_FW IQ change (lag 4): 0.16–0.20 NTU	all cases: 7% [3; 12]	1, 4, 6–7 ^†^, 7–9 ^†^, 8, 10, 13	Tu_FW **◊	Schwartz et al., 1997 [10]
Philadelphia (USA)	1992–1993	1.2 M	Admissions to hospital	>64	2-3-3	Rivers	9–19 (100–150) ^d^	20–40 (1000)	PCh, CFD, RFi, pChm	0.17–0.20	6021	Tu_FW IQ change (lag 9–11): 0.16–0.21 NTU	all cases: 9% [5; 13]	4–6 ^†^, 9, 10, 11	Tu_FW **	S2: Schwartz et al., 2000 [27]
Edmonton (Canada)	1993–1998	845,000	Admissions to hospital, visits to emergency department, visits to GP	All, 2–18, 19–65, >65	1-2-3	River	35 (1500)	400 (15,000)	CFD, Rfi, Ch, pChm	0.04 (0.38)	62,060				Precipitation, Tu_RW, coliforms in raw water, Tu_FW, particle count, air temperature, change in the location of water abstraction point (0/1)	Lim et al., 2003 [28]
Québec (Canada)	2000–2002	240,000	Calls for medical advice	All	1-1-1	River	1.7–3.2	62 (340)	PCh, CFD, Rfi, Oz, pCh	0.27 (0.75)	3555	Tu_FW daily change from min. to max.: 0.11–0.75 NTU	33–76% depending on the lag	11, 15, 17	Tu_FW **, precipitation	Gilbert et al., 2006 [29]
Atlanta (USA)	1993–2004	3.0 M	Visits to emergency department	All	3-9-8	Rivers	Hourly max: 1.5–55 (1984)	100 (1000) ^e^	CFD, RFi, Ch (UV for 3 DWTPs), pCh	0.03–0.17	240,925	IQ change in Tu_FW: 0.04–0.09 NTU (lags 4–6)10 NTU change in Tu_RW over three weeks	0.5% [−0.2; 1.2] (NS except for 1/8 distribution zones: 6% [4; 8])	4–11	Tu_RW ***	Tinker et al., 2010 [30]
Nantes (France)	2002–2007	410,000	Consultations of GP	<16, >15	1-1-1	River	20 (124)	120 (7000)	CFD, Rfi, Oz, pCh	0.05 (0.35)	103,149	Tu_FW IQ change (lags 7–9): 0.04–0.06 NTU	4.2% [1.5; 6.9] (child.), 2.9 [0.5; 5.4] (ad.)	7–15	Precipitation, Tu_RW, Tu_FW **◊, air temperature ***◊, river flow ***◊, produced flow **◊, free chlorine, interventions for broken pipe *, hydrant flushes	Beaudeau et al., 2014 [31]
Gothenburg (Sweden)	2008–2011	500,000	Calls for medical advice	All	2-1-1	River and lake	5 (40)	36 (6500)	CFD, Rfi, Ch	<0.05	25,659	40 mm precipitation in 24 h (lag 5)	17% [7; 27]	4–7	Precipitation **, number of consecutive dry days, number of consecutive wet days **	Tornevi et al., 2013 [32]
Paris-Est (France)	2002–2007	379,000	Consultations of GP	<16, >15	2-2-1	Rivers	15–16 (124–149)	6200–6700 (125,000–240,000)	[CFD], RFi or Flot, Sfi, Oz, pCh (syst. 1) ; CoagRFi, Sfi, Oz, pCh (syst. 2)	0.03–0.05 (0.14–0.19)	99,315	Tu_FW P10-P50 change (lags 6–8): 0.03–0.04 NTU	13% [4; 18] (child.), 14% [4; 16] (ad.)	6–8 ^†^	Precipitation, Tu_RW **, Tu_FW **◊, water temperature ***◊, river flow **◊, produced flow *, free chlorine	Rambaud et al., 2014 [16]
Paris area—Nord (France)	2002–2007	673,000	Consultations of GP	<16, >15	1-2-1	River	20 (147)	1600 (40,000)	CFD, RFi, Oz, NanoFi, UV, pCh (DWTP 1); CFD, RFI, Oz, Ch (DWTP 2)	0.04 (0.05)	246,165	IQ change of particle count in filtered water (lags 6–8): 147–333 units/mL (0.03–0.05 NTU)	ERR = 12.1% [7.5; 17.0] (child.), 8.5% [4.3; 12.9] (ad.)	6–8 ^†^, 5–13	Tu_RW, turbidity in filtered water *, particle count in finished water ***◊, TOC in raw water ***◊, water temperature ***◊, river flow ***◊, proportion of nanofiltered water, produced flow ***◊	Rambaud et al., 2015 [17]
Paris area—Est (France)	2002–2007	874,000	Consultations of GP	<16, >15	1-1-1	River	30 (320)	3100 (48,000)	CFD, RFI, Oz, Ch	0.04 (0.05)	322,773	IQ change in particle count in filtered water (lags 6–8): 52–150 units/mL (0.04–0.04 NTU)	NS		Tu_RW, turbidity in filtered water **, Tu_FW, particle count in filtered water, TOC in raw water ***◊, TOC in filtered water, water temperature ***◊, river flow ***◊, produced flow ***	Rambaud et al., 2015 [17]
Paris area—Sud (France)	2002–2007	1.4 M	Consultations of GP	<16, >15	1-1-1	River	16 (220)	1600 (43,000)	CFD, RFI, Oz, Ch	0.03 (0.14)	375,613	IQ change in particle count in filtered water (lags 6–8): 25–65 units/mL (0.03–0.03 NTU)	ERR = 3.8% [1.0; 6.7] (child.), 2.7% [−0.3; 5.7] (ad.)	6–10	Tu_RW *, turbidity in filtered water **, Tu_FW, particle count in filtered water ***◊, TOC in raw water **◊, TOC in filtered water *, water temperature ***◊, river flow *◊, produced flow ***	Rambaud et al., 2015 [17]
Nancy (France)	2002–2007	247,000	Consultations of GP	<16, >15	1-2-1	River	8 (290)	2,000 (16,000)	PCh, CFD, Oz, pCh	0,07 (0,23)	87,007	Tu_FW IQ change (lags 5–7): 0.06–0.08 NTU	NS		Tu_RW, Tu_FW, water temperature ***◊, river flow, produced flow **, water cuts, interventions for broken pipe	Rambaud et al., 2016 [18]
Vancouver (Canada)	1992–1998	2.1 M	Visits to GP, admissions to hospital	All, <16, >64	3-3-3	Reservoirs	0.5–1.3 (8–19)	1–2 (38–51)	Ch	0.5–1.3 (8–19)	14,571 H admission; 1.102 M visits to GP	Tutbidity > 1 NTU	Attributable Risk: 0.8–2.1 visits to GP; 0.2–1.3% H visits	3–6 ^†^, 6–9 ^†^, 12–16 ^†^, 21–29 ^†^	Turbidity ***◊, precipitation, fecal coliform	Aramini et al., 2000 [33]
Boston (USA)	1998–2008	1.5 M	Visits to hospital	>64	1-1-1	Reservoirs	0.34 (0.68)	1.5 (43)	Ch/Oz, pChm	0.34 (0.68)	36,456	Turbidity IQ change (lags 8–12): 0.28–0.39 NTU	ERR = 3.7% [1.2; 6.3]	8–12 ^†^, 13–17 ^†^, 18–22 ^†^, 23–27 ^†^, 28–32 ^†^, 33–37 ^†^	Precipitation, turbidity corrected from algae **, water temperature **, fecal coliforms *, cyanobacteria *, ozone *, abs.UV350, CT	Beaudeau et al., 2014 [34]
New York (USA)	2002–1999	9.2 M	Visits to emergency department	All, 1–4, 5–17	3-3-1	Reservoirs	0.98–1.0 (2.80–2.85)	1–2 (14–57) ^f^	Ch	0.97 (2.38)	438,000	Turbidity IQ change (lag 6): NA	5% [3;6] in spring, NS in other seasons	3–11	Turbidity ***◊ (only in spring)	Hsieh et al., 2015 [35]
Le Havre (France)	1994–1996, 1997–2000	80,000	Drug sales	All	2-2-1	Karstic springs	4 (>200) (syst. 1); 0.1 (1) (syst. 2)	80 (1000) (syst. 1); 8 (50) (syst. 2)	[CFD], Rfi, Ch (syst. 1); Ch (syst. 2)	0.3 (>1.5) (syst. 1); 0.1 (1.0) (syst. 2)	14,600 drug boxes (2500 cases)	IQ change in Tu_FW over lags 6–8:0.13–0.27 NTU (syst. 1); 0.08–0.11 NTU (syst. 2)	2.8 [−0.6;7.2] (syst. 1); 9.4 [5.2; 13.7] (syst. 2)	6–8 ^†^, 9–10 (syst. 2), 13–15 (syst. 1)	Precipitation, turbidity ** (syst. 2), Tu_RW (syst. 1), Tu_FW* (syst. 1), produced flow, free chlorine (hourly min.) ** (syst. 2), decantation * (syst. 1)	Beaudeau et al. 2012 [36]
Angoulême (France)	2002–2007	50,000	Consultations of GP	<16, >15	1-1-1	Karstic spring	4 (27)	31 (1700)	CoagRFi, Ch	0.14 (2)	21,336	P10-P50 change in Tu_RW over lags 7–9: 1.1–2.9 NTU	30% [0; 60] (child.), 15% [−15; 45] (ad.)	7–9 ^†^, 13–15 ^†^	Precipitation, Tu_RW *, Tu_FW, air temperature *◊, produced flow *◊, interventions for broken pipe *◊	Rambaud et al., 2013a [14]
Paris-Centre (France)	2002–2007	160,000	Consultations of GP	<16, >15	3-1-1	Karstic springs	0.08–0.23 (0.50–0.73)	1–8 (14–150)	Ch	0.17 (0.66)	26,526	IQ change in Tu_FW over lags 7–9: 0.11–0.22 NTU	11.8% [1.2; 22.5] (child.), 4.1% [−0.2; 8.8] (ad.)	7–9 ^†^, 10–11	Precipitation, turbidity **◊, air temperature ***, free chlorine, produced flow *◊, contribbution of the most fecally contaminated resource in the produced flow *	Rambaud et al., 2013b [15]

^a^ Treatment facilities: PCh: Pre-chlorination; CFD: Coagulation-floculation-decantation; [CFD]: CFD operated if high turbidity; RFi: Rapid filtration; CoagRFi: Coagulation and Rfi; SFi: Slow filtration; NanoFi: Nanofiltration; Oz: Ozone disinfection; Ch: Chlorine disinfection; UV: UV-disinfection; pCh: Post-chlorination; pChm: Post-chloramination; Syst.: System: couple (resource + DWTP). ^b^ Significant lags: *p* < 0.05; ^†^: Combined lags. ^c^ Significance and robustness of the exposure-AGE risk functions: * *p* < 0.1; ** *p* < 0.01; *** *p* < 0.001; ◊ Association reproduced in different populations, age classes, or with different health indicators. Consulted websites for raw water quality: ^d^ [37]; ^e^ [38]; ^f^ [39].

**Table 2:** Indicators tested as exposure proxies in the selected time series studies (TSS).

Indicator	Shape of the Risk Function	Commentary	Frequency of Positive Tests ^a^	Sites
Concentration of fecal coliform or *Escherichia coli* in raw water	Increasing, linear	Lack of sensitivity to viral or protozoan contamination.	1/3	Edmonton, Boston *, Vancouver
Cyanobacteria	Increasing, linear	Possibly relevant for reservoir waters in the absence of clarification facilities.	1/1	Boston *
Turbidity in finished water (in the presence of clarification facilities)	Increasing	Suspended particles may carry pathogens. May indicate resource contamination and/or treatment transient weaknesses. May interact with river flow.	5/12	Philadelphia *, Edmonton, Québec *, Atlanta, Nantes *, Paris-Est *, PA-Nord, PA-Est, PA-Sud, Nancy, Le Havre * (syst. 1), Angoulême
Particle count in filtered/finished water (in the presence of clarification facilities)	Increasing	Alternative to turbidity in finished water. More precise when turbidity is very low.	2/4	Edmonton, PA-Nord *, PA-Est, PA-Sud *
Turbidity in raw/finished water (in the absence of clarification facilities)	Increasing	The availability of algae data makes possible to correct turbidity from algae influence (Boston). May interact with water temperature or season.	5/5	Vancouver *, Boston *, New York *, Le Havre (sys. 2) *, Paris-Centre *
Turbidity in raw water (in the presence of clarification facilities)	Increasing	May better correlate to AGE than turbidity in finished water.	5/10	Atlanta *, Edmonton, Nantes, Paris-Est *, PA-Nord, PA-Est, Paris-Sud *, Nancy, Le Havre (sys. 1) *, Angoulême *
Precipitation	Increasing with threshold	Alternative to turbidity in raw water.	1/10	Edmonton, Québec, Nantes, Paris-Est, Gothenburg *, Vancouver, Boston, Le Havre, Angoulême, Paris-Centre
Numbers of consecutive days of wet weather	Increasing	Derived from precipitation. Surrogate for wetness of soils (facilitating surface runoff).	1/1	Gothenburg *
Numbers of consecutive days of dry weather	No expectation	Derived from precipitation. Unclear.	1/1	Gothenburg
total organic carbon (TOC) in raw water	No expectation	Unclear. May interact with river flow.	3/3	PA-Nord *, PA-Est *, PA-Sud *
total organic carbon (TOC) in filtered water	No expectation	Unclear.	1/2	PA-Est, PA-Sud *
River flow	U-shaped	High or low flows may be associated to fecal pollution. Heavy precipitations bring about both high river flows and river contaminations. Low flows result in less dilution of urban effluents. May modify the turbidity risk function.	5/6	Nantes *, Paris-Est *, PA-Nord *, PA-Est *, PA-Sud *, Nancy
Water temperature	U-shaped	High or low temperature may enhance the AGE risk (via waterborne or other route exposure), possibly depending on climate. May modify the turbidity or TOC -AGE association.	6/6	Paris-Est *, PA-Nord *, PA-Est *, PA-Sud *, Nancy *, Boston *
Air temperature	U-shaped	Beside a possible direct and synchronous effect on health care pursue (Boston and New York), may also serve as a surrogate to water temperature (exposure).	3/4	Edmonton, Nantes *, Angoulême *, Paris-Centre *
Produced flow	U-shaped or increasing	Sub optimal operation conditions at low or high produced flow.	8/9	Nantes *, Paris-Est *, PA-Nord *, PA-Est *, PA-Sud *, Nancy *, Le Havre, Angoulême *, Paris-Centre *
CT (disinfectant concentration × time of contact)	Decreasing	Measure of the disinfection power; available in the USA.	0/1	Boston
Free chlorine concentration at the outlet of the treatment plant	Decreasing with threshold	Hourly minimum may be relevant to highlight a risk associated to transient breakdowns, if direct distribution (i.e., no buffer effect of storage).	1/4	Nantes, Le Havre *, Paris-Est, Paris-Centre
Permanent change in abstraction or treatment facilities (Boolean)	Improvement	E.g., change in abstraction point, implementation of ozonation instead of chlorination.	1/2	Edmonton, Boston * (respectively)
Episodic change in treatment (Boolean)	Improvement	Decantation implementation interacts with turbidity on AGE incidence.	1/1	Le Havre *
Daily number of water cuts	Increasing, linear	Adverse impact limited to the inhabitants next downstream of the intervention point. TSS are poorly adequate to address this risk.		Nancy
Daily number of interventions for broken pipe	Increasing, linear	Idem.	2/3	Nantes *, Nancy, Angoulême *
Daily number of hydrant flushings	Increasing, linear	Idem.	0/1	Nantes
Daily number of consumers’ complaints	Increasing	Idem. Additional limitation: few complaints are specific to fecal contamination.	0/1	Nantes

^a^ Number of sites with positive test/number of sites testing the indicator. *: Site with positive test (i.e., meeting significance (*p*-value < 0.10) and plausibility criteria).

## Abbreviations

AGE	Acute gastroenteritis
CFD	Coagulation-flocculation-decantation
DWS	Drinking water system
DWTP	Drinking water treatment plant
DZ	Distribution zone
ERR	Excess of relative risk
GAM	Generalized additive model
GP	General Practitioner
ICD	International classification of diseases
IQ	Interquartile
NTU	Nephelometric turbidity unit
P10	10th Percentile
PA	Paris area
PRISMA	Preferred reporting items for Systematic Reviews and Meta-Analysis
TRF	Turbidity risk function
TSS	Time series study
Tu_FW	Turbidity of finished water
Tu_RW	Turbidity of raw water
USA	United States of America
UV	Ultraviolet
